# Neural Stem Cell-Derived Exosomes Regulate Neural Stem Cell Differentiation Through miR-9-Hes1 Axis

**DOI:** 10.3389/fcell.2021.601600

**Published:** 2021-05-13

**Authors:** Ping Yuan, Lu Ding, Huili Chen, Yi Wang, Chunhong Li, Shu Zhao, Xiaoyu Yang, Yizhao Ma, Jie Zhu, Xinrui Qi, Yanyan Zhang, Xiaohuan Xia, Jialin C. Zheng

**Affiliations:** ^1^Center for Translational Neurodegeneration and Regenerative Therapy, Tenth People’s Hospital of Tongji University, Shanghai, China; ^2^Department of Cardio-Pulmonary Circulation, School of Medicine, Shanghai Pulmonary Hospital, Tongji University, Shanghai, China; ^3^Translational Research Institute of Brain and Brain-Like Intelligence, Shanghai Fourth People’s Hospital affiliated to Tongji University School of Medicine, Shanghai, China; ^4^Collaborative Innovation Center for Brain Science, Tongji University, Shanghai, China

**Keywords:** exosome, miRNA, miR-9, Hes1, differentiation, maturation, neural stem cells

## Abstract

Exosomes, a key element of the central nervous system microenvironment, mediate intercellular communication via horizontally transferring bioactive molecules. Emerging evidence has implicated exosomes in the regulation of neurogenesis. Recently, we compared the neurogenic potential of exosomes released from primary mouse embryonic neural stem cells (NSCs) and astrocyte-reprogrammed NSCs, and observed diverse neurogenic potential of those two exosome populations *in vitro*. However, the roles of NSC-derived exosomes on NSC differentiation and the underlying mechanisms remain largely unknown. In this study, we firstly demonstrated that NSC-derived exosomes facilitate the differentiation of NSCs and the maturation of both neuronal and glial cells in defined conditions. We then identified miR-9, a pro-neural miRNA, as the most abundantly expressed miRNA in NSC-derived exosomes. The silencing of miR-9 in exosomes abrogates the positive effects of NSC-derived exosomes on the differentiation of NSCs. We further identified *Hes1* as miR-9 downstream target, as the transfection of Hes1 siRNA restored the differentiation promoting potential of NSC-derived exosomes after knocking down exosomal miR-9. Thus, our data indicate that NSC-derived exosomes facilitate the differentiation of NSCs via transferring miR-9, which sheds light on the development of cell-free therapeutic strategies for treating neurodegeneration.

## Introduction

Neurodegenerative diseases, including Alzheimer’s disease (AD), Parkinson’s disease (PD), and Huntington’s disease (HD), are a heterogeneous group of disorders that display the progressive neurodegeneration in specific regions of the central nervous system (CNS), leading to the function abnormalities and disabilities. Among neurodegenerative diseases, especially age-related ones, the impairment of neurogenesis is one key pathological feature (Steiner et al., 2006; Zhang et al., 2007). Due to the failure of clinical trial of drugs for eliminating key risk factors (e.g., Aβ) of neurodegenerative disorders, to maintain and expand the neural stem cell (NSC) pool and to facilitate the regenerative potential of NSCs have been considered as a promising therapeutic strategy for treating these diseases (Steiner et al., 2006; Abdipranoto et al., 2008). The stemness and differentiation potential of NSCs are regulated by cell extrinsic factors in the NSC niche (Yang, 2004; Kageyama et al., 2019; Ahmad et al., 2020). For example, during early CNS development, Notch signaling keeps NSCs uncommitted via activating its intercellular effectors, HES and HEY transcriptional repressor families (Tomita et al., 1996; Engler et al., 2018). Notch signaling also work in concert with SHH and Wnt pathways to facilitate NSCs proliferation (Engler et al., 2018; Ahmad et al., 2020). Thus, the temporal patterning of aforementioned pathways in the NSC niche controls NSC maintenance, differentiation, and cell lineage commitment (Noelanders and Vleminckx, 2017; Engler et al., 2018). Emerging evidence has implicated exosomes as a key part of the NSC niche (Zhang et al., 2017; Ma et al., 2019). Exosomes, a key mediator of intercellular communication, are small bilipid layer-enclosed extracellular vesicles (30–150 nm) that regulate various physiological and pathological processes through horizontally transferring bioactive cargos among cells (Valadi et al., 2007; Ramachandran and Palanisamy, 2012; Xia et al., 2019b). Zhang et al. reported that hypothalamic NSC-derived exosomes significantly slowdown aging-mediated hypothalamic NSC loss through transferring exosomal miRNAs (Zhang et al., 2017). Due to the potential effects of exosomes in the regulation of NSCs, the application/administration of stem cell-derived exosomes as a novel approach to stimulate endogenous neurogenesis (Oh et al., 2017; Yang et al., 2017). For instance, systemic administration of multipotent mesenchymal stromal cell (MSC)-derived exosomes effectively improves functional recovery by promoting endogenous angiogenesis and neurogenesis in rats after traumatic brain injury (TBI) (Xin et al., 2013; Zhang et al., 2015). MSC-derived exosomes, loaded with microRNAs (miRNAs), such as miR-124 or miR-17∼92, improve neurological function via enhancing the neuronal identity of cortical NSCs in TBI and ischemia animal models (Xin et al., 2017; Yang et al., 2017, 2019). Exosomes derived from human umbilical vein endothelial cells (HUVEs) also promote the proliferation and stemness maintenance of NSCs, displaying a potential to expand NSC pool during brain regeneration (Zhang et al., 2018).

Although great progress has been made to demonstrate the roles of exosomes in neurogenesis and neuroregeneration, multiple knowledge gaps remain there to be filled. For example, we are still in lack of information including but not limited to the effects and underlying mechanisms of embryonic NSC-derived exosomes (EXOs) on the regulation of NSCs and the potential interplay between EXOs and diverse signaling pathways in the NSC niche. To address those questions, we for the first time reported the involvement of the EXOs in the regulation of embryonic NSCs in defined conditions (Ma Y. et al., 2018; Ma et al., 2019). Interestingly, although EXOs have no significant effects on the proliferation of NSCs, those exosomes promote the generation of neurons from NSCs (Ma Y. et al., 2018; Ma et al., 2019). Our findings unveil the neurogenic potential of EXOs, however, the exact roles of EXOs in NSC differentiation and the underlying mechanisms remain largely unknown. To investigate the effects of EXOs on NSCs, in the current study, we co-cultured mouse embryonic NSCs with EXOs, and observed positive effects of EXOs on the fate commitment of NSCs and maturation of differentiated cells. We then examined the abundance of miRNAs in EXOs through microarray- and RT-qPCR-based approaches, and identified miR-9 as the most highly enriched one. We further demonstrated the essential roles of miR-9 in NSC differentiation by either directly manipulating the expression of miR-9 in NSCs or silencing exosomal miR-9 in the EXO-NSC co-culture system. Lastly, we identified the key downstream target of exosomal miR-9, *Hes1*, since silencing *Hes1* restored the differentiation promoting potential of miR-9-deleted EXOs. Our study demonstrated an important role of EXOs in the regulation of NSCs and dissected the underlying molecular mechanisms, which, provides the theories foundation for the amplification of EXOs in treating neurodegenerative diseases.

## Materials and Methods

### Isolation and Enrichment of Mouse NSCs

NSCs were isolated from mouse fetal brain tissue as previously described (Ma K. et al., 2018). Briefly, cortical tissues were isolated from embryonic day 13.5 (E13.5) C57BL/6J mice and triturate physically 15–20 times. Dissociated tissues were filtered through 40 μm filter. Single cells were cultured in substrate-free tissue culture flasks for the formation of neurospheres in NSC proliferation medium, containing NeuroCult^®^ NSC Basal Medium (Stem Cell Technologies), NeuroCult^®^ NSC Proliferation Supplements (Stem Cell Technologies), 20 ng/mL FGF2 (BioWalkersville), 20 ng/mL EGF (BioWalkersville), and 2 μg/mL heparin (Sigma), N2 supplement (Gibco), 2 mM L-glutamine (ThermoFisher), 100 IU/mL penicillin (ThermoFisher), and 100 μg/mL streptomycin (ThermoFisher). Primary neurospheres were collected, centrifuge at low speed to remove flowing cells, dissociated into single cells by accutase (Sigma) for 5 min at 37°C, and re-plated for a second round of neurosphere formation. Enriched NSCs were harvested after three rounds of neurosphere formation.

### Differentiation of NSCs

The differentiation of NSCs was carried out as previously described (Ma et al., 2019). Briefly, 5 × 10^3^ NSCs were planted on Matrigel-coated coverslips in 24-well plate with DMEM/F12 (Gibco) supplemented with 1 × N2 supplement (Gibco), 1 × B27 supplement (Gibco), 1.0 mM GlutaMAX (ThermoFisher), 10 ng/mL brain-derived neurotrophic factor (BDNF) (Peprotech), 10 ng/mL glial cell line-derived neurotrophic factor (GDNF) (Peprotech), and 2% Knockout Serum Replacement (Gibco). The medium was changed every 2 days.

### Collection of Exosomes

Exosomes were isolated from the culture medium of NSCs as previously described (Ma Y. et al., 2018). Briefly, 6 × 10^6^ NSCs were plated in poly-L-Ornithine/laminin-coated 10 cm dish and cultured in NSC proliferation medium for 12 h. The supernatants were collected and exosomes were collected by gradient centrifugation: supernatants were first centrifuged at 300 g for 10 min to remove flowing cells, at 3,000 g for 20 min to remove cellular debris, at 10,000 g for 30 min to remove intracellular organelles and then at 100,000 g for 2 h to precipitate exosomes. All steps of centrifugation were handled at 4°C. Exosomal protein concentrations were determined with a BCA Protein Assay Kit (Pierce). For PKH67 labeling, every 100 μg exosomes were incubated with 2 nmol PKH67 for 10 min at room temperature (RT). Exosomes were re-collected through ultra-speed centrifugation.

### Agonist/Antagonist/siRNA and Transfection

The agomiR control, agomiR-9, antagomiR control, antagomiR-9, siRNA scrambled control, and Hes1 siRNA were purchased from GenePharma (GenePharma). Transfection of 20 nM agomiR-9/antagomiR-9/Hes1 siRNA or their corresponding controls was performed using the Lipofectamine 2000 reagent (Invitrogen) according to the manufacturer’s instruction.

### Transmission Electron Microscopy

Negative staining of exosome suspensions followed by imaging in a transmission electron microscope was used to determine vesicle shape and size distribution. Aliquots of exosome suspensions were dispensed onto sheets of Parafilm in a humidified petri dish and the vesicles were deposited on carbon-coated grid (300-mesh) for 3 min. Subsequently, the grid was negatively stained with 1% uranyl acetate for 3 min and excess stain was blotted off. The droplets of exosomes were removed with filter paper and air-dried at RT. Images were taken by transmission electron microscopy (JEM-1230, JEOL).

### Nanoparticle Tracking Analysis

The size and number of exosomes were carried out as previously described (Ma Y. et al., 2018). Briefly, isolated EVs were resuspended in 150 μL PBS and diluted at 1:100 in PBS. 1 mL solution was used for NTA that was assessed on NanoSight NS300 system (Malvern Instruments) with a sCMOS camera. The conditions of the measurements were set at 25°C, 1 cP viscosity, 25 s per capture frame and 60 s measurement time. Three individual measurements were applied for determining the size and concentration of exosomes.

### Nano-Flow Cytometry

The size and number of exosomes were identified by NanoFCM. NanoFCM is applicable when the refractive index of input samples are the same or similar to that of silica particles. The standard working curve of scattering light intensity is established using silica standard sphere. EVs isolated from 50 mL conditioned medium were resuspended in 100 μL PBS for NanoFCM. The particle size distribution of exosome samples is measured based on the scattering intensity.

### Immunocytochemistry

Differentiated NSCs were fixed in 4% formaldehyde for 20 min at RT and then washed with PBS for three times. The fixed cells were permeabilized with 0.2% Triton X-100 in PBS for 10 min, blocked with 2% BSA in PBS for 1 h at RT, and then incubated overnight at 4°C with primary antibodies including Map2 (rabbit, Sigma, 1:200), βIII-Tubulin (Tuj1) (mouse, Millipore, 1:200), Glast (rabbit, Abcam, 1:100), and GFAP (chick, CST, 1:200). Coverslips were washed with PBS for three times and incubated for 1 h at RT with secondary antibodies including anti-rabbit IgG (coupled with Alexa Fluor 568, Life Technologies), anti-rabbit IgG (coupled with Alexa Fluor 488, Life Technologies), anti-chicken IgG (coupled with Alexa Fluor 488, Life Technologies), and anti-mouse IgG (coupled with Alexa Fluor 488, Life Technologies). Coverslips were mounted using VectaShield (Vector Laboratories) and images were taken by a Zeiss AX10 fluorescence microscope accompanied with ZEN 2.3 (blue edition) software. For quantification of the percentage of specific cell types in each experiment, cell type-specific antigen positive cells were counted from 15 random fields per group in three coverslips (five fields each).

### Quantitative Reverse Transcription-Polymerase Chain Reaction

The mRNA and miRNA were isolated from cell samples using RNeasy mini kit (Qiagen) according to the manufacturer’s instructions. Genomic DNA was removed and cDNA was synthesized using DNase I digestion kit (Qiagen) and miScript II reverse transcription kit (Qiagen), respectively. Transcripts were amplified using gene-specific primer (Supplemental Table 1) and SYBR green PCR kit (Qiagen) with the ABI7500 (Applied Biosystems). All RT-qPCR results measured each sample in triplicate and no-template blanks were used for negative controls. Amplification curves and gene expression were normalized to the house-keeping gene *Gapdh* (for mRNA) and *U6* snRNA (for miRNA).

### Western Blotting

Western blotting was performed as previously described (Gao et al., 2019). Exosomes were lysed in RIPA lysis and extraction buffer (ThermoFisher) containing a protease inhibitor cocktail (Sigma). Protein concentrations were determined with a BCA Protein Assay Kit (Pierce). Proteins (20–30 mg) were separated by sodium dodecyl sulfate polyacrylamide gel electrophoresis (SDS-PAGE) and electrophoretic transferred to polyvinylidene fluoride membranes (Millipore and Bio-Rad). Membranes were incubated with primary antibodies for CD9 (rabbit, Abcam, 1:2,000), Flottlin1 (mouse, BD Biosciences; 1:5,000), TSG (rabbit, Abcam, 1:1,000), APOA1 (rabbit, Affinity Biosciences; 1:500), APOA2 (rabbit, Affinity Biosciences; 1:500), Hes1 (rabbit, Affinity Biosciences; 1:500), and β-actin (mouse, CST; 1:1,000) overnight at 4°C followed by a secondary anti-rabbit or anti-mouse antibody (Cell Signaling Technologies, 1:10,000) incubation. Antigen-antibody complexes were visualized by Pierce ECL Western Blotting Substrate (ThermoFisher). For data quantification, films were scanned with a CanonScan 9950F scanner; the acquired images were analyzed using ImageJ program.

### MicroRNAs Microarray

Total RNA was extracted from EXOs and 3 μg total RNA per sample was used as input material for the small RNA library. Sequencing libraries were generated using NEBNext^®^ Multiplex Small RNA Library Prep Set for Illumina^®^ (NEB). The clustering of the index-coded samples was performed on a cBot Cluster Generation System using TruSeq SR Cluster Kit v3-cBot-HS (Illumia). After cluster generation, the library preparations were sequenced on an Illumina Hiseq 2,500/2,000 platform and 50 bp single-end reads were generated. Raw data (raw reads) of fastq format were firstly processed through custom perl and python scripts for quality control. The small RNA tags were mapped to reference sequence by Bowtie without mismatch to analyze their expression and distribution on the reference. Mapped small RNA tags were used to looking for known miRNA. miRBase 20.0 was used as reference for known miRNA, miRDeep2, and sRNA-tools-cli were used to obtain novel miRNAs and draw the secondary structures, respectively. miRNA expression levels were estimated by transcript per million (TPM).

### Statistical Analyses

All results are the means of at least three independent experiments ± SE. The statistical difference between two independent groups was analyzed with the unpaired Student’s *t*-test, and that among more than two groups was assessed with the parametric one-way ANOVA with *post hoc* Bonferroni test. Significance was considered when *p* < 0.05.

## Results

### EXOs Promote the Differentiation of NSCs

**FIGURE 1:**
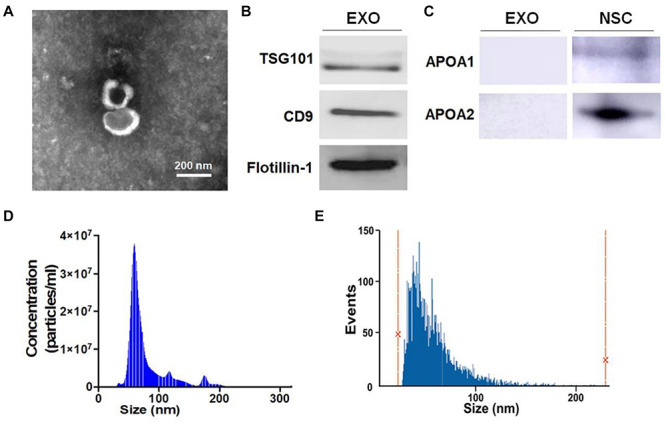
Characterization of exosomes (EXOs). **(A)** Purified exosomes were observed under transmission electron microscopy (TEM) using negative staining. **(B)** The levels of positive exosomal markers TSG101, CD9, and Flotillin-1 in protein lysates of neural stem cells (NSC)-derived exosome pellets were determined by western blotting. **(C)** The levels of negative exosomal markers APOA1 and APOA2 in protein lysates of NSC-derived exosome pellets and NSCs were determined by western blotting. **(D,E)** Particle-size distribution of exosomes was determined by NanoSight analysis (NTA) in panel **(D)** and NanoFCM in panel **(E)** technologies. Scale bar 200 nm in panel **(A)**.

To test the effects of EXOs on the differentiation of NSCs, we isolated and characterized ultracentrifugation-enriched EXOs. TEM visualized the cup-shaped appearance of exosomes with sizes less than 200 nm (Figure 1A). Western blotting analysis detected strong expression of three positive protein markers of exosomes including TSG101, CD9, and Flotillin-1, in the collected exosome samples (Figure 1B). Additionally, negative protein markers APOA1 and APOA2 were expressed in NSC lysate but not in the exosome samples, confirming the purity of exosomes (Figure 1C). Both NTA and NanoFCM analyses further confirmed the typical size distribution of ultracentrifugation-enriched EXOs (30–150 nm), confirming the purification of EXOs (Figures 1D,E).

**FIGURE 2:**
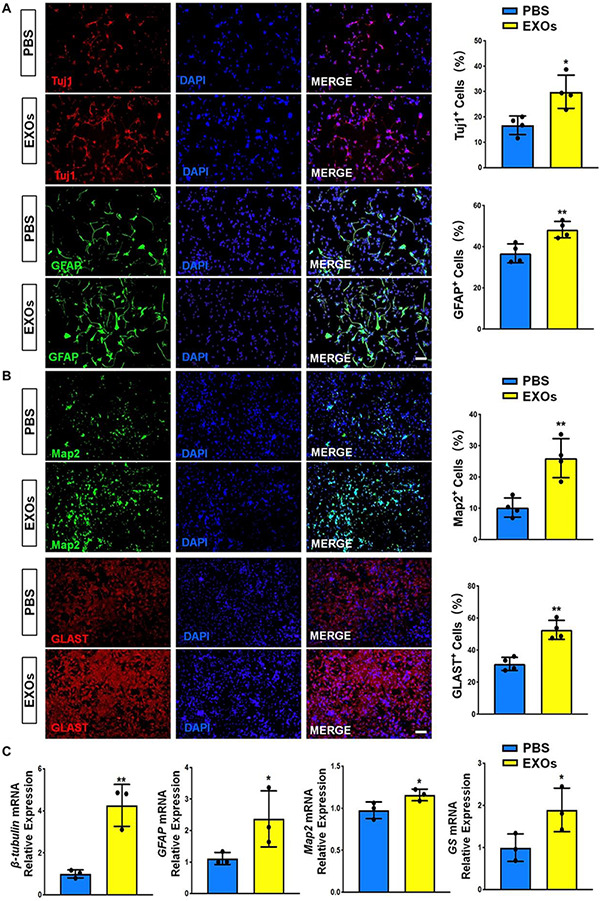
Exosomes promote NSC differentiation. **(A)** NSCs were co-cultured with exosomes for 6 days in differentiation conditions. Representative images of pre-mature neuronal (Tuj1) and glial (GFAP) markers staining were shown. Proportions of cells expressing pre-mature cell-specific immunoreactivities were determined (in the right panel). **(B)** Representative images of matured neuronal (Map2) and glial (Glast) staining were shown. Proportions of cells expressing matured cell-specific immunoreactivities were determined (in the right panel). **(C)** The transcript expression of pre-mature cell markers (β*III-tubulin* and *GFAP*) and matured cell markers (*Map2* and *GS*) was determined by RT-qPCR analysis. Data were represented as mean ± SE from three independent experiments. * and ** denote *p* < 0.05 and *p* < 0.01, respectively. Scale bar 100 μm in panel **(A,B)**.

The internalization of exosomes by NSCs was validated by incubating PKH67-labeled exosomes with primary NSCs for 12 h (Supplementary Figure 1). We then co-cultured NSCs with 15 μg/mL EXOs in differentiation conditions for 6 days. The immunofluorescence analysis suggested that EXOs enhanced NSC differentiation, ascertained by higher proportions of Tuj1^+^ neuronal and GFAP^+^ glial cells in exosome-treated groups versus PBS controls (Figure 2A). Next, we examined the effects of EXOs on neuronal and glial maturation. The immunofluorescence analysis indicated that more matured neurons (Map2^+^ cells) and astrocytes (Glast^+^ cells) were presented in EXO-treated groups, compared to PBS controls (Figure 2B). RT-qPCR analysis also revealed an increase in the levels of transcripts corresponding to pre-mature neuronal markers (β*III-tubulin*), pre-mature astroglial markers (*GFAP*), matured neuronal markers (*Map2*) and matured astroglial markers (*GS*) in EXO-treated group, compared to PBS controls, confirming the immunostaining results (Figure 2C). The effects of EXOs on NSC differentiation were further confirmed by co-culturing EXOs with NSCs in differentiation conditions for 3 and 9 days (Supplementary Figures 2, 3). Thus, our observations suggested that EXOs facilitate the differentiation of NSCs and the maturation of both neuronal and glial cells.

**FIGURE 3:**
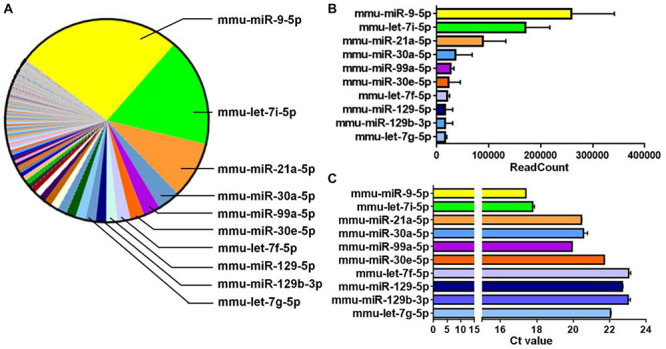
miR-9 is predominantly expressed in EXOs. **(A)** The microRNAs (miRNAs) profiles of NSC-derived exosomes were determined by miRNA microarray and a parts of whole table for the readcounts of all detected miRNAs was generated. The 10 miRNAs with the most readcounts were marked on the right. **(B)** The readcounts of the top 10 most abundantly expressed miRNAs. Data were represented as mean ± SD from three independent biological samples. **(C)** The Ct value of RT-qPCR analysis for the top 10 most abundantly expressed miRNAs, identified by miRNA microarray, in NSC-derived exosomes. Data were represented as mean ± SE from three independent experiments.

### miR-9 Is Abundantly Expressed in EXOs

To dissect the mechanisms underlying the positive effects of EXOs on differentiation and maturation, we examined the exosomal miRNA profile through miRNA microarray. 565 mouse miRNAs have been identified by microarray. Among all detected miRNAs, miR-9 displayed the highest abundance (Figure 3A) and readcount (TPM) (Figure 3B). This result was validated by RT-qPCR that, of the top 10 miRNAs with the highest readcounts, miR-9 exhibited lowest Ct value (Figure 3C). Both results indicate the highest abundance of miR-9 in EXOs.

### miR-9 Positively Regulates NSC Differentiation

**FIGURE 4:**
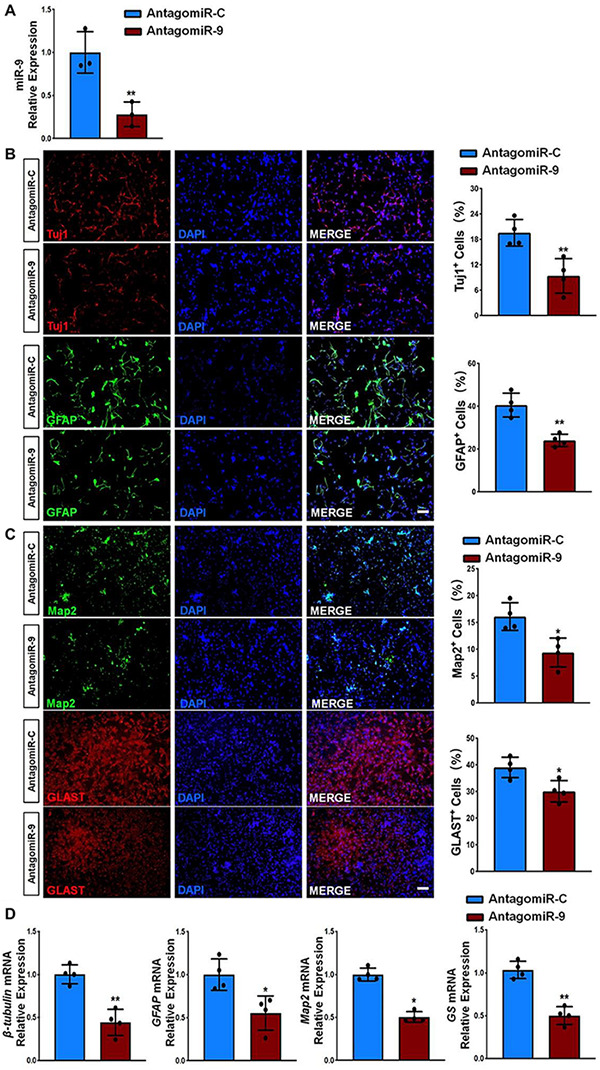
miR-9 loss-of-function inhibits NSC differentiation. **(A)** NSCs transfected with either antogomiR-C or antogomiR-9 were cultured for 6 days in differentiation conditions. The transfection efficiency was determined by quantifying the intracellular miR-9 expression levels via RT-qPCR analysis. **(B)** Representative images of pre-mature cell markers (Tuj1 and GFAP) staining were shown. Proportions of cells exhibiting immunoreactivities of pre-mature cell markers (Tuj1^+^ and GFAP^+^) were determined (in the right panel). **(C)** Representative images of matured cell markers (Map2 and Glast) staining were shown. Proportions of cells exhibiting immunoreactivities of pre-mature cell markers (Map2^+^ and Glast^+^) were determined (in the right panel). **(D)** The expression levels of transcripts corresponding to pre-mature cell markers (β*III-tubulin* and *GFAP*) and matured cell markers (*Map2* and *GS*) was determined by RT-qPCR analysis. Data were represented as mean ± SE from three independent experiments. * and ** denote *p* < 0.05 and *p* < 0.01, respectively. Scale bar 100 μm in panel **(B,C)**.

Interestingly, multiple reports have implied the neurogenic roles of *miR-9* (Coolen et al., 2012, 2013). To confirm it, we examined the effects of miR-9 on NSC differentiation by perturbation-of-function approaches using specific antagonist and agonist, antagomiR-9 and agomiR-9, respectively. NSCs were firstly transfected with either miR-9 antagonist (antagomiR-9) or its corresponding control (antagomiR-C) and cultured in differentiation conditions for 6 days. The knockdown efficiency was validated by RT-qPCR, where significant down-regulation of miR-9 expression levels was observed in antagomiR-9 group, compared to antagomiR-C group (Figure 4A). Immunofluorescence analysis demonstrated a significant decrease in the proportions of both Tuj1^+^ and GFAP^+^ cells once we inhibited miR-9 expression during the differentiation of NSCs (Figure 4B). Furthermore, we also observed a significant decrease in the proportions of both Map2^+^ and Glast^+^ cells in antagomiR-9 group versus controls (Figure 4C). Our findings were corroborated by RT-qPCR analysis that revealed a significant decrease in the expression levels of transcripts corresponding to pre-mature cell markers (β*III-tubulin* and *GFAP*) and matured cell markers (*Map2* and *GS*), in antagomiR-9 group versus controls (Figure 4D). Thus, our results indicate that the lack of miR-9 blocks or delays the cell fate commitment of NSCs and the maturation of differentiated cells.

**FIGURE 5:**
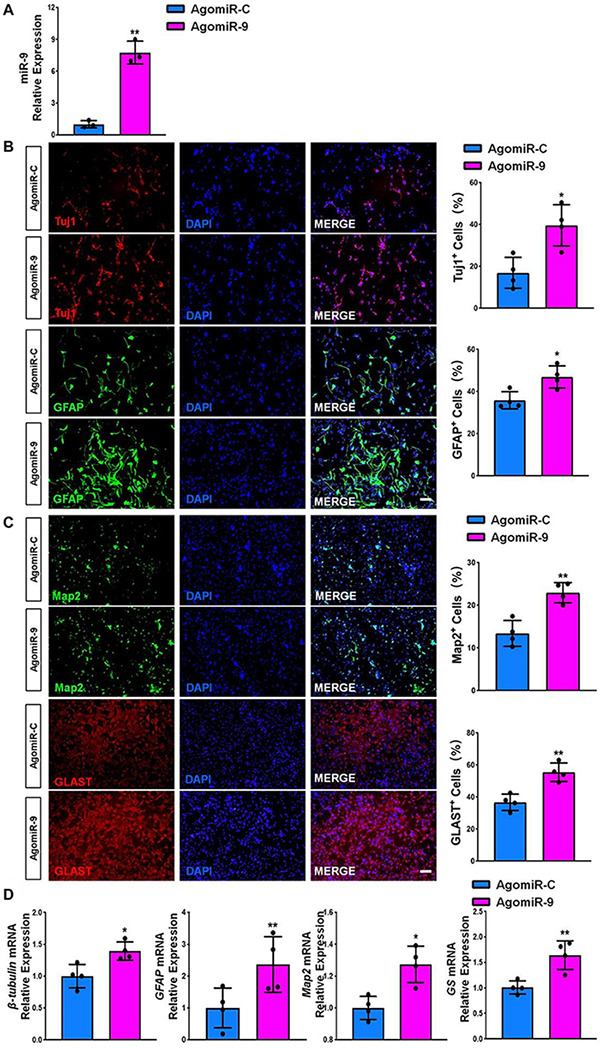
miR-9 gain-of-function promotes NSC differentiation. **(A)** NSCs transfected with either agomiR-C or agomiR-9 were cultured for 6 days in differentiation conditions. The transfection efficiency was determined by quantifying the intracellular miR-9 expression levels via RT-qPCR analysis. **(B)** Representative images of pre-mature cell markers (Tuj1 and GFAP) staining were shown. Proportions of cells exhibiting immunoreactivities of pre-mature cell markers (Tuj1^+^ and GFAP^+^) were determined (in the right panel). **(C)** Representative images of matured cell markers (Map2 and Glast) staining were shown. Proportions of cells exhibiting immunoreactivities of pre-mature cell markers (Map2^+^ and Glast^+^) were determined (in the right panel). **(D)** The expression levels of transcripts corresponding to pre-mature cell markers (β*III-tubulin* and *GFAP*) and matured cell markers (*Map2* and *GS*) was determined by RT-qPCR analysis. Data were represented as mean ± SE from three independent experiments. * and ** denote *p* < 0.05 and *p* < 0.01, respectively. Scale bar 100 μm in panel **(B,C)**.

Next, we transfected NSCs with either miR-9 agonist (agomiR-9) or control agonist (agomiR-C). Transfected cells were then cultured in differentiation conditions for 6 days. AgomiR-9 transfection significantly increased the expression levels of miR-9 in NSCs (Figure 5A). The proportions of both Tuj1^+^/Map2^+^ neurons and GFAP^+^/Glast^+^ astrocytes also increased significantly in agomiR-9 group, compared with agomiR-C group (Figures 5B,C). Additionally, the transcript levels of pre-mature cell markers (β*III-tubulin* and *GFAP*) and matured cell markers (*Map2* and *GS*) were similarly increased in agomiR-9 group versus controls, validated by RT-qPCR analysis (Figure 5D). Taken together, our results demonstrated *that miR-9 functions* as an important regulator in enhancing the differentiation of NSCs and the maturation of both neurons and glia.

### Exosomes Regulate NSC Differentiation via miR-9

**FIGURE 6:**
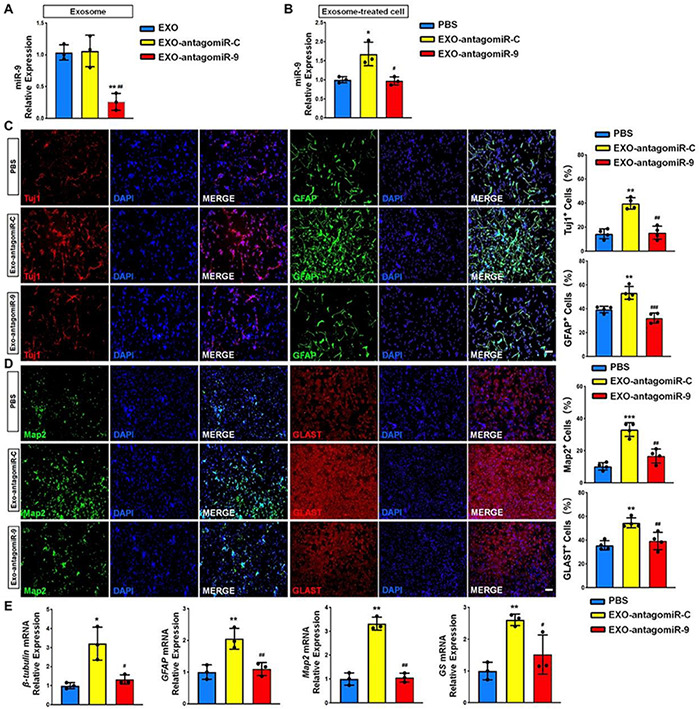
miR-9 mediates the effects of EXOs on NSC differentiation. **(A)** NSCs were transfected with either antogomiR-C or antogomiR-9. The knockdown of miR-9 expression levels in exosomes derived from antogomiR-9-transfected NSCs were validated by RT-qPCR. **(B)** NSCs treated with PBS, EXO-antagomiR-9, or EXO-antagomiR-C were cultured for 6 days in differentiation conditions. The expression levels of miR-9 in NSCs treated with PBS or exosomes were determined by RT-qPCR analysis. **(C)** Representative images of pre-mature cell markers (Tuj1 and GFAP) staining were shown. Proportions of cells exhibiting immunoreactivities of pre-mature cell markers (Tuj1^+^ and GFAP^+^) were determined (in the right panel). **(D)** Representative images of matured cell markers (Map2 and Glast) staining were shown. Proportions of cells exhibiting immunoreactivities of pre-mature cell markers (Map2^+^ and Glast^+^) were determined (in the right panel). **(E)** The expression levels of transcripts corresponding to pre-mature cell markers (β*III-tubulin* and *GFAP*) and matured cell markers (*Map2* and *GS*) were determined by RT-qPCR analysis. Data were represented as mean ± SE from three independent experiments. * and ** denote *p* < 0.05 and *p* < 0.01 in comparison to control, respectively. # and ## denote *p* < 0.05 and *p* < 0.01 in comparison to EXO-antagomiR-C group, respectively. Scale bar 100 μm in panel **(C,D)**. ***p < 0.001, ^###^*p* < 0.001.

To determine whether the positive effects of EXOs on NSC differentiation is mediated by miR-9, we transfected NSCs with either antagomiR-9 or antagomiR-C using the approach described above and collected exosomes in the culture medium 48 h post transfection. RT-qPCR analysis revealed that the expression levels of miR-9 was significantly reduced in exosomes derived from antagomiR-9 transfected NSCs (EXO-antagomiR-9), compared to EXOs and antagomiR-C-transfected NSCs (EXO-antagomiR-C) (Figure 6A). NSCs were then co-cultured with either EXO-antagomiR-9 or EXO-antagomiR-C under differentiation conditions for 6 days. RT-qPCR analysis revealed that the expression levels of miR-9 were significantly increased in NSCs co-cultured with EXO-antagomiR-C, compared with PBS controls (Figure 6B). Moreover, no difference in miR-9 expression was observed between EXO-antagomiR-9-treated and PBS control groups. The immunofluorescence analysis demonstrated that the positive influence of EXO-antagomiR-C on NSC differentiation was abrogated by depleting exosomal miR-9, determined by the quantification of Tuj1^+^ neurons and GFAP^+^ astrocytes (Figure 6C). In addition, the positive effects of EXO-antagomiR-C on cell maturation was compromised by knocking down exosomal miR-9, ascertained by the significant decrease in the proportions of Map2^+^ neurons and Glast^+^ astrocytes in EXO-antagomiR-9 group versus EXO-antagomiR-C group (Figure 6D). The expression levels of transcripts corresponding to pre-mature cell markers (β*III-tubulin* and *GFAP*) and matured cell markers (*Map2* and *GS*) were also significantly reduced in EXO-antagomiR-9 group, compared to EXO-antagomiR-C group (Figure 6E). Together, our results suggested that miR-9 is the key cargo in mediating the effects of EXOs on the differentiation of NSCs and the maturation of both neurons and glia.

### Exosomes Regulate NSC Differentiation via miR-9-Hes1 Axis

**FIGURE 7:**
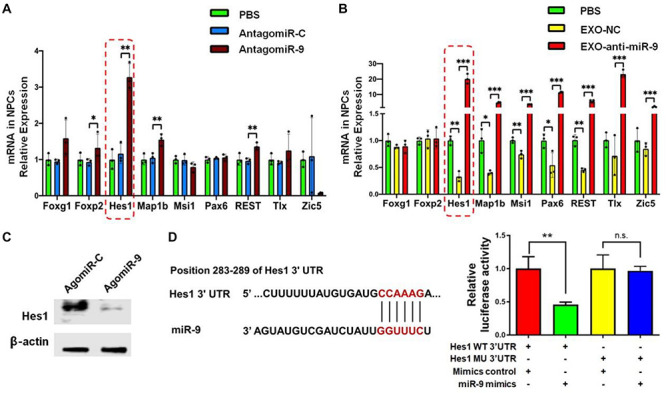
*Hes1* expression is negatively regulated by miR-9 in NSCs. **(A)** The expression levels of transcripts corresponding to known targets of miR-9 in NSCs transfected with either antagomiR-9 or antagomiR-C were determined by RT-qPCR analysis. **(B)** The expression levels of transcripts corresponding to known targets of miR-9 in NSCs co-cultured with either EXO-antagomiR-9 or EXO-antagomiR-C were determined by RT-qPCR analysis. **(C)** Representative western blotting results showing the expression of Hes1 and β-actin proteins in either agomiR-C- or agomiR-9-transfected NSCs. **(D)** The predicted consequential pairing of *Hes1* 3′UTR (top) and miR-9 (bottom) on the TargetScan website (left). Repression of luciferase activities by the *Hes1* 3′UTR were dependent on miR-9 (right). Firefly luciferase activities were normalized to the internal control, Renilla luciferase activities. Data were represented as mean ± SE from three independent experiments. ^∗^, ^∗∗^, and ^∗∗∗^ denote *p* < 0.05, *p* < 0.01, and *p* < 0.001, respectively. ns, non-significance in comparison to control.

To gain insight into the mechanism underlying exosomal miR-9 influence on NSC differentiation, we examined the expression patterns of known miR-9 target genes including *Foxg1*, *Foxp2*, *Hes1*, *Map1b*, *Msi1*, *Pax6*, *REST*, *Tlx*, and *Zic5* (Clovis et al., 2012; Coolen et al., 2013; Radhakrishnan and Anand, 2016). RT-qPCR results demonstrated that the expression levels of *Hes1*, *Map1b*, *REST*, and *Foxp2* transcripts were up-regulated significantly in antagomiR-9-transfected NSCs, compared to both PBS and antagomiR-C controls (Figure 7A). RT-qPCR analysis also found that the knockdown of miR-9 in EXOs reversed the negative effects of EXOs on the expression levels of *Hes1*, *Map1b*, and *REST* transcripts, but not that of *Foxp2* transcripts in NSCs (Figure 7B). Furthermore, *Hes1* transcript levels displayed the largest fold changes among aforementioned three genes, therefore, we chose *Hes1* as exosomal miR-9 target candidate for following studies. We then carried out western blotting to confirm the inverse correlation between *Hes1* and miR-9 expression. Down-regulation of Hes1 protein levels was observed in agomiR-9-transfected NSCs versus controls (Figure 7C). The direct interaction between miR-9 and *Hes1* 3′ untranslated region (UTR) was validated by dual luciferase assay. Co-transfection of agomiR-9 and Dual-Luciferase reporter constructs containing the wild-type *Hes1* 3′UTR, but not that containing miR-9 target site mutated *Hes1* 3′UTR, significantly decreased the firefly activity in HEK293A cells, normalized by the Rellina activity, indicating miR-9 directly targets *Hes1* (Figure 7D).

**FIGURE 8:**
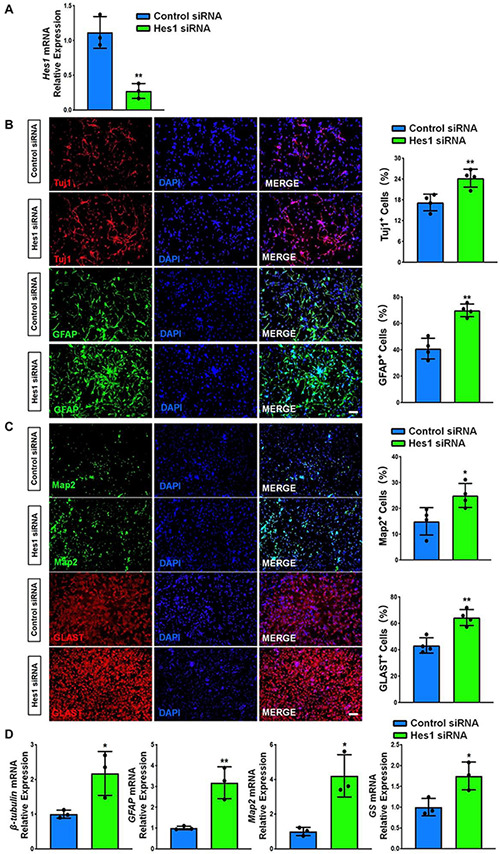
*Hes1* loss-of-function enhances NSC differentiation. **(A)** NSCs transfected with either Hes1 siRNA or scrambled control were cultured for 6 days in differentiation conditions. The transfection efficiency was determined by quantifying the intracellular *Hes1* expression levels via RT-qPCR analysis. **(B)** Representative images of pre-mature cell markers (Tuj1 and GFAP) staining were shown. Proportions of cells exhibiting immunoreactivities of pre-mature cell markers (Tuj1^+^ and GFAP^+^) were determined (in the right panel). **(C)** Representative images of matured cell markers (Map2 and Glast) staining were shown. Proportions of cells exhibiting immunoreactivities of pre-mature cell markers (Map2^+^ and Glast^+^) were determined (in the right panel). **(D)** The expression levels of transcripts corresponding to pre-mature cell markers (β*III-tubulin* and *GFAP*) and matured cell markers (*Map2* and *GS*) was determined by RT-qPCR analysis. Data were represented as mean ± SE from three independent experiments. * and ** denote *p* < 0.05 and *p* < 0.01, respectively. Scale bar 100 μm in panel **(B,C)**.

We carried out loss-of-function study to address the effects of Hes1 on NSC differentiation. NSCs were transfected with either Hes1 siRNA or scrambled control (control siRNA) and cultured in differentiation conditions for 6 days. The knockdown efficiency was validated by RT-qPCR, where significant reduction of *Hes1* expression levels was observed in Hes1 siRNA group, compared to control siRNA group (Figure 8A). Immunofluorescence analysis demonstrated a significant increase in the proportions of both Tuj1^+^ and GFAP^+^ cells once Hes1 expression was inhibited during NSC differentiation (Figure 8B). Furthermore, higher proportions of both Map2^+^ and Glast^+^ cells were observed in Hes1 siRNA group versus control siRNA group (Figure 8C). Our findings were confirmed by RT-qPCR analysis which revealed a significant elevation in the expression levels of transcripts corresponding to pre-mature cell markers (β*III-tubulin* and *GFAP*) and matured cell markers (*Map2* and *GS*) in Hes1 siRNA group versus control siRNA group (Figure 8D). Both immunofluorescence and RT-qPCR analyses revealed Hes1 as an important repressor of NSC differentiation.

**FIGURE 9:**
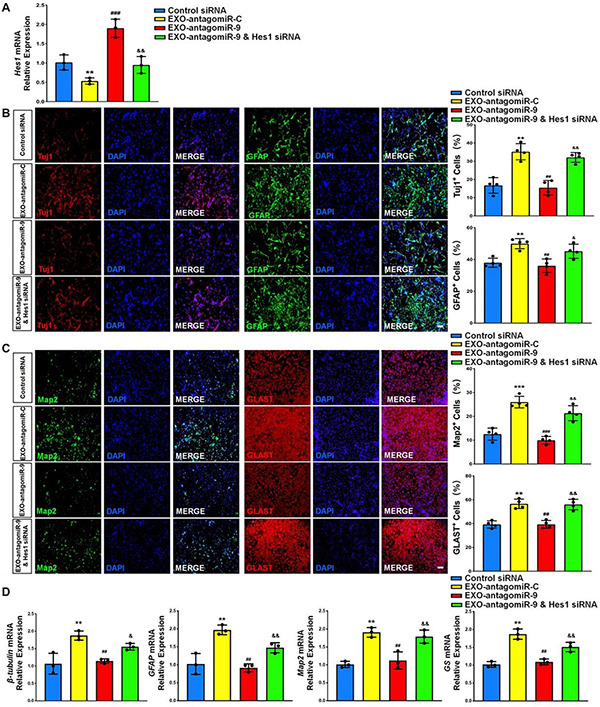
Exosomal miR-9 regulates NSC differentiation via repressing *Hes1*. **(A)** NSCs were divided into four groups for scrambled siRNA transfection, EXO-antogomiR-C co-culture, EXO-antogomiR-9 co-culture, or EXO-antogomiR-9 co-culture with Hes1 siRNA transfection. NSCs were then cultured in differentiation conditions for 6 days. The expression levels of *Hes1* in each group were determined by RT-qPCR. **(B)** Representative images of pre-mature cell markers (Tuj1 and GFAP) staining were shown. Proportions of cells exhibiting immunoreactivities of pre-mature cell markers (Tuj1^+^ and GFAP^+^) were determined (in the right panel). **(C)** Representative images of matured cell markers (Map2 and Glast) staining were shown. Proportions of cells exhibiting immunoreactivities of pre-mature cell markers (Map2^+^ and Glast^+^) were determined (in the right panel). **(D)** The expression levels of transcripts corresponding to pre-mature cell markers (β*III-tubulin* and *GFAP*) and matured cell markers (*Map2* and *GS*) were determined by RT-qPCR analysis. Data were represented as mean ± SE from three independent experiments. *, **, and *** denote *p* < 0.05, *p* < 0.01, and *p* < 0.001 in comparison to control, respectively. #, ##, and ### denote *p* < 0.05, *p* < 0.01, and *p* < 0.001 in comparison to EXO-antagomiR-C group, respectively. & and && denote *p* < 0.05 and *p* < 0.01 in comparison to EXO-antagomiR-9 group, respectively. Scale bar 100 μm in panel **(C,D)**.

At last, we investigated whether or not Hes1 acts as the downstream target of exosomal miR-9 during NSC differentiation. NSCs were co-cultured with either EXO-antagomiR-9 or EXO-antagomiR-C. A subgroup in EXO-antagomiR-9 group was co-transfected with Hes1 siRNA to inhibit Hes1 expression. NSCs treated with PBS of the same volume as exosome suspension and then transfected with scrambled siRNA were utilized as control group. NSCs in all groups were cultured in differentiation conditions for 6 days. RT-qPCR analysis demonstrated that the reduction of *Hes1* transcript expression in EXO-antagomiR-C groups was abrogated by knocking down miR-9 in EXOs (Figure 9A). The upregulation of *Hes1* transcript expression in EXO-antagomiR-9 groups was further eliminated by Hes1 siRNA treatment, validating the transfection efficiency of Hes1 siRNA. Quantification of cell type-specific markers revealed that the silencing of Hes1 significantly restored the proportions of pre-mature Tuj1^+^ neurons and GFAP^+^ astrocytes (Figure 9B). Besides, the proportions of matured cells (Map2^+^ neurons and Glast^+^ astrocytes) were similarly restored after down-regulating Hes1 expression in EXO-antagomiR-9 group (Figure 9C). RT-qPCR results also demonstrated similar patterns that the Hes1 repression by siRNA significantly enhance the expression levels of transcripts corresponding to pre-mature cell markers (β*III-tubulin* and *GFAP*) and matured cell markers (*Map2* and *GS*) in EXO-antagomiR-9 group (Figure 9D). Taken together, these results suggested that *Hes1* transcripts were targeted by exosomal miR-9-mediated repression for facilitating the differentiation of NSCs and the maturation of both neurons and glia.

## Discussion

Neurodegeneration is the progressive neuronal atrophy and loss-of-function, which is present in various neurodegenerative diseases. The transplantation of stem cells with regenerative capacity has shown great promise for treating these diseases (Wang et al., 2007; Kim et al., 2015). Previous studies from us and other groups show that only a small proportion of transplanted cells survive and differentiate into neurons (Li et al., 2001; Tian et al., 2015). Recent evidence has suggested that stem cells participate in brain remodeling and functional recovery by paracrine effect rather than cell replacement, since stem cell-secreted exosomes elicit similar biological activity to the stem cells themselves (Camussi and Quesenberry, 2013; Zhang et al., 2015). Post administration, these exosomes achieve their regenerative function majorly through promoting endogenous neurogenesis and angiogenesis (Zhang et al., 2015, 2018). Currently, multiple types of stem cells including MSCs, HUVEs, embryonic stem cells have been utilized to study the feasibility of exosome-based cell free therapeutic strategy, and among them, MSCs are the most commonly investigated one (Zhang et al., 2015). Unlike the aforementioned types of stem cells, NSCs are the cell sources that directly generate neurons and neuroglia in the brain, implying EXOs may exhibit strong neurogenic potential. Recent studies showed that EXOs alleviate mitochondrial damage and synaptic dysfunction in cortical neurons of AD mouse (Li et al., 2020). However, the effects of EXOs on neurogenesis during CNS development remain vague. We previously reported the important roles of EXOs in regulating embryonic NSC proliferation and differentiation (Ma Y. et al., 2018; Ma et al., 2019). In this study, we followed our previous work and demonstrated that EXOs enhance the differentiation of NSCs and the maturation of both neuronal and glial cells in defined conditions. miRNA microarray and RT-qPCR analyses identified miR-9 as the most abundantly expressed miRNAs in EXOs. The perturbation-of-function approaches further confirmed the important role of miR-9 in the regulation of NSCs. And last, we showed that the positive effects of EXOs on NSC differentiation could be abrogated by depleting exosomal miR-9. Thus, our study unveils a possible mechanism for the EXO-mediated NSC differentiation.

Brain development follows a precise temporal and spatial patterning, which requires complicated regulatory network for the proper regulation of NSC in both embryonic and post-natal stages. In our study, we collected NSCs from the cortical tissue of mouse embryos at E13.5, when robust neurogenesis takes place *in vivo* (Semple et al., 2013). Since the majority of neurons and glia have not been differentiated and matured during early CNS development, the self-regulation is an important aspect for NSC regulation (Semple et al., 2013). Besides classic signaling pathways, our study indicates EXOs as an important element of NSC niche. Through secreting exosomes, NSCs enhance their commitment, facilitating the proper generation of neurons and glia during brain development. It is worth-noting that multiple single cell RNA-seq data have reveals NSCs are heterogeneous during embryonic neurogenesis (Zhong et al., 2018). NSCs can divide symmetrically and asymmetrically to generate NSCs and differentiated cells at the same time (Gotz and Huttner, 2005). It raises an interesting question that whether all NSCs secrete EXOs to promote the differentiation of entire NSC population or only a sub-population of NSCs secrete EXOs to promote the differentiation of another NSC sub-population. The answer of this question can further explain extend our understanding of the roles of EXOs in the regulation of NSCs, which is currently under investigation.

miR-9, the most abundant expressed EXO miRNA, is a key regulator of proper timing of neurogenesis (Radhakrishnan and Anand, 2016). During development, miR-9 is one of the most highly expressed miRNAs in the early and adult vertebrate brain (Kapsimali et al., 2007; Bonev et al., 2011, 2012; Radhakrishnan and Anand, 2016). Shibata et al. further demonstrated that miR-9 is enriched in proliferative zone in telencephalon, which is widely involved in regulating proliferation, maturation, and differentiation of neurons (Bonev et al., 2011, 2012; Shibata et al., 2011). Our observations further corroborate the importance of miR-9 in regulating NSC differentiation through perturbation-of-function approaches. Several studies have suggested that the exosomal miRNA expression signatures are cell type-dependent (Baumgart et al., 2017; Ma et al., 2019). For instance, high-throughput screening and ectopic expression approaches showed that the intracellular levels of free miRNAs significantly influence the exosomal miRNA profile (Squadrito et al., 2014; Ma et al., 2019). Embryonic NSCs express high levels of miR-9 (data not shown), as miR-9 is required to maintain their neurogenic competence. It explains, partially at least, the high expression levels of cellular and exosomal miR-9. It is also worth-noting that multiple active mechanisms for sorting miRNAs into exosomes were discovered recently. In these active sorting processes, RNA-binding proteins including nSMase2 (Kosaka et al., 2013), hnRNPA2B1 (Villarroya-Beltri et al., 2013), and AGO2 (McKenzie et al., 2016) were recruit to transport miRNAs with specific motifs into exosomes. However, there is no evidence that implies the specific binding of miR-9 with these proteins, leaving the protein-based sorting of exosomal miR-9 as an open question for future investigation.

Currently, miR-9 has been proved to target multiple genes during brain development and NSC differentiation including *Hes1*, *REST*, *Zic5*, *Foxg1*, *Foxp2*, *Pax6*, *Msi1*, *Tlx*, and *Map1b* (Clovis et al., 2012; Coolen et al., 2013; Radhakrishnan and Anand, 2016). In our system, *Hes1* is the gene that demonstrates the largest fold change after co-culturing NSCs with EXOs. Hes1 is a basic helix-loop-helix transcriptional repressor that promotes the maintenance of NSCs and gliogenesis by inhibiting pro-neural gene expression (Tan et al., 2012). Being activated by Notch signaling pathway, Hes1 down-regulates *Ascl1*, *Ngn2*, and other pro-neural genes to block neurogenesis, maintaining the proper timing of neural tube development (Hatakeyama et al., 2004). Surprisingly, our observations suggest that, in a defined condition, miR-9-Hes1 axis may equally modulate neurogenesis and gliogenesis at the same time, instead of regulating the cell fate commitment toward different lineages. Our results are supported by others’ studies that investigate the effects of Hes1 on human NSC differentiation (Yang et al., 2020). Thus, our finding, together with others’ observations (Kobayashi and Kageyama, 2010; Mendez-Maldonado et al., 2018; Yang et al., 2020), indicates that the involvement of Notch signaling and *Hes1* in NSC regulation is highly time- and condition-dependent. During CNS development, Hes1 acts as a key neural fate determinant in early stage and then functions as an anti-neural regulator in post-natal stage (Kobayashi and Kageyama, 2010; Mendez-Maldonado et al., 2018). Furthermore, Hes1 has been reported to negatively regulate Notch signaling in a feedback manner during CNS development (Kobayashi and Kageyama, 2010; Boareto et al., 2017). In our study, we have observed the significant up-regulation of key Notch signaling components *Dll1*, *Notch1*, and *Notch2* expression in Hes1 down-regulated NSCs (Supplementary Figure 4). Our results imply that the Hes1 loss-of-function may induce a compensation of Notch activity that overcomes the influence of Hes1 knockdown on gliogenesis in our model. Besides, we found that exosomal miR-9 also negatively regulated the expression levels of *REST*, and *Map1b*. REST is a neuronal repressor that facilitates the generation of glial cells from NSCs (Xia et al., 2019a). Map1b is a key protein in enhancing axonal growth and branching by stabilizing axonal microtubules (Bouquet et al., 2004). Our results suggest EXOs and exosomal miR-9 may serve as a general promoter of differentiation, instead of regulating the cell fate commitment toward certain lineage. Thus, although we cannot exclude the involvement of REST and Map1b in the exosomal miR-9-mediated neurogenesis, it is highly likely that both REST and Map1b are not the main downstream effectors of exosomal miR-9. Thus, our results identify exosome-mediated miR-9 transferring as a powerful and effective approach, other than direct surface contact (e.g., Notch signaling pathway) and soluble factor diffusion (e.g., Wnt and Shh signaling pathway), in intercellular communication that regulates neurogenesis.

Besides, mounting evidence implicates exosomes as a perfect natural drug delivery system for treating CNS disorders (Alvarez-Erviti et al., 2011; Haney et al., 2015). Except for regular small molecule drugs, miRNAs that may possess therapeutic potential by targeting multiple risk genes were recruited in these pioneer studies, and miR-124, a well-known pro-neural miRNA, is the most widely used one (Xia et al., 2019b). For example, miR-124a-loaded exosomes that were secreted by MSCs or HEK293 cells significantly enhance adult neurogenesis post ischemia and TBI (Yang et al., 2017), repress *REST* expression in Huntington’s disease mouse model (Lee et al., 2017), promote the polarization of microglia into anti-inflammatory phenotype under neuroinflammatory conditions (Yang et al., 2019), and reduce in viability or clonogenicity of glioma cells (Lang et al., 2017). Although miR-124 and miR-9 have different seed sequences, they are considered as two central miRNAs in controlling neuron fate and synaptic morphology (Stappert et al., 2015; Xue et al., 2016). The important neurogenic functions of miR-9 make it a promising candidate for exosome-based delivery in treating neurodegeneration and enhancing neuroregeneration, especially in accelerating adult neurogenesis *in vivo*, which will be examined in our future works.

**FIGURE 10:**
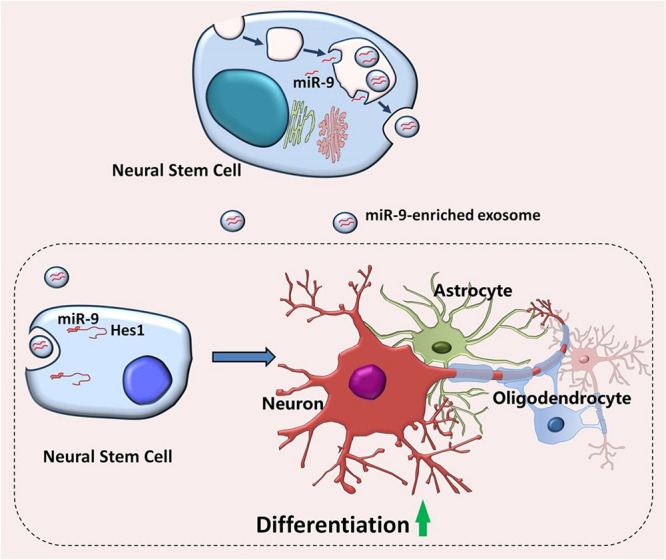
Proposed model of EXO-mediated regulation of NSCs. Embryonic NSCs secrete exosomes enriched with miR-9. After internalizing by neighboring NSCs, exosomes release miR-9 into the recipient cells, leading to the repression of differentiation repressor gene, Hes1. The inhibition of Hes1 then facilitates the differentiation of NSCs and the maturation of both neuronal and glial cells.

In summary, our study demonstrated the abundant expression of miR-9, a key regulator of proliferation and neuronal differentiation, in EXOs (Figure 10). We further showed that the positive effects of EXOs on NSC differentiation are mediated by miR-9 and its downstream gene *Hes1*. Thus, our study, combining with our previous reports, provides a possible mechanism for the exogenous NSC-mediated modification of microenvironment in favor to differentiation and neurogenesis, shedding light on the development of exosome-based cell free therapeutic strategies to activate adult neurogenesis *in vivo*.

## Data Availability Statement

The raw data supporting the conclusions of this article will be made available by the authors, without undue reservation.

## Ethics Statement

The animal study was reviewed and approved by The Institutional Animal Care and Use Committee of Tongji University School of Medicine.

## Author Contributions

JZhe and XX designed the experiments. PY, LD, HC, CL, SZ, XY, and YM performed the experiments. PY, XX, YW, CL, JZhu, XQ, and YZ analyzed the data. XX, PY, YW, and JZhe prepared the manuscript. All authors read and approved the final manuscript.

## Conflict of Interest

The authors declare that the research was conducted in the absence of any commercial or financial relationships that could be construed as a potential conflict of interest.

## Abbreviations

AD: Alzheimer’s disease
CNS: central nervous system
E13.5: embryonic day 13.5
EXOs: neural stem cell-derived exosomes
HD: Huntington’s disease
HUEVs: human umbilical vein endothelial cells
miRNA: microRNA
MSC: mesenchymal stromal cell
NanoFCM: nano-flow cytometry
NSC: neural stem cell
NTA: nanoparticle tracking analysis
PD: Parkinson’s disease
RT: room temperature
RT-qPCR: quantitative reverse transcription-polymerase chain reaction
SDS-PAGE: sodium dodecyl sulfate polyacrylamide gel electrophoresis
TBI: traumatic brain injury
TEM: transmission electron microscopy
TPM: transcript per million.
