# Supplementary material for: Neural Stem Cell-Derived Exosomes Regulate Neural Stem Cell Differentiation Through miR-9-Hes1 Axis
Source: Front Cell Dev Biol. 2021 May 13;9:601600. doi: 10.3389/fcell.2021.601600 (PMC8155619; doi:10.3389/fcell.2021.601600)
Supplement: Supplementary file 1 [file Data_Sheet_1.docx]

**Supplemental Information**

**Neural stem cell-derived exosomes regulate neural stem cell differentiation through miR-9-Hes1 axis**

**Ping Yuan, Lu Ding, Huili Chen, Yi Wang, Chunhong Li, Shu Zhao, Xiaoyu Yang, Yizhao Ma, Jie Zhu, Xinrui Qi, Yanyan Zhang, Xiaohuan Xia, Jialin C. Zheng**

**Supplemental Materials**

Supplementa Figure 1

Supplemental Figure 2

Supplemental Figure 3

Supplemental Figure 4

Supplemental Table 1


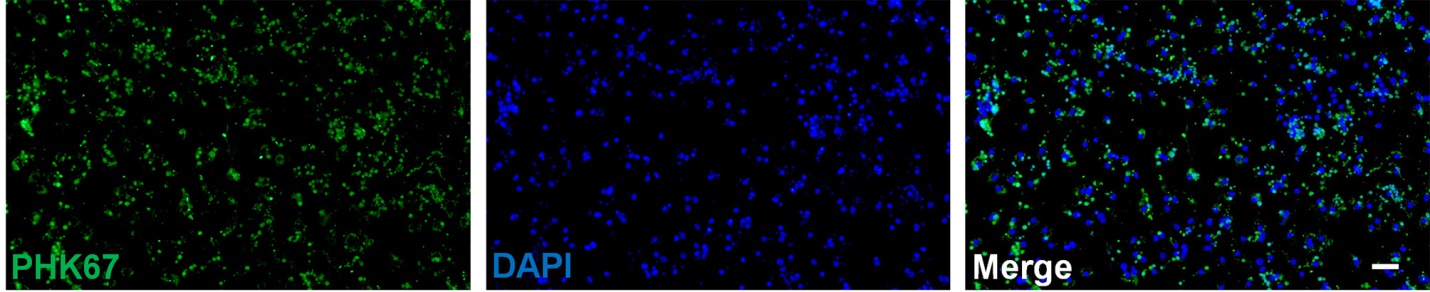


**Supplementary Figure 1. NSCs internalize EXOs.**

The uptake of PKH67-labeled EXOs by NSCs was determined by immunocytochemical analysis.


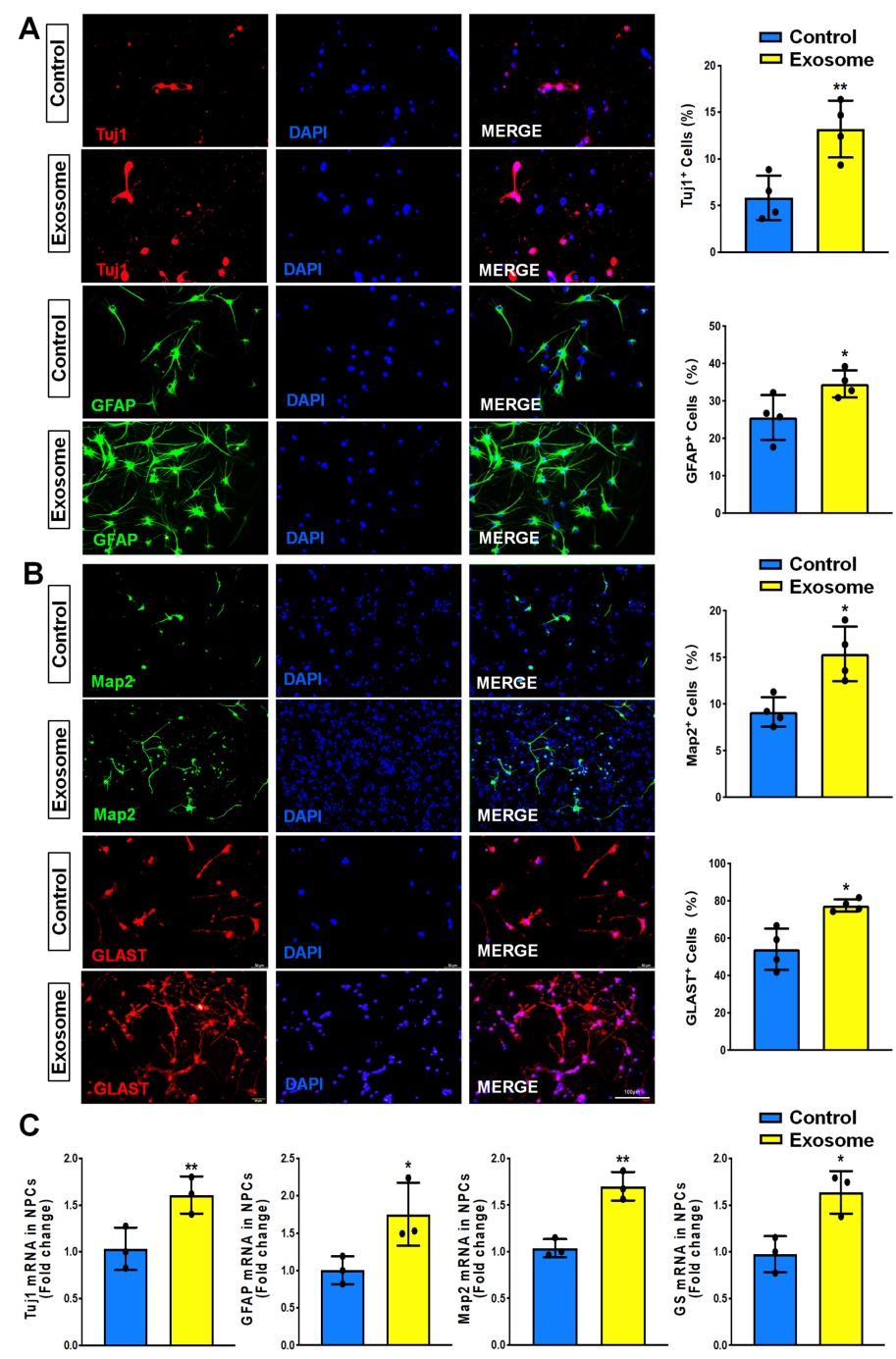


**Supplementary Figure 2. EXOs promote NSC differentiation at culture day 3.**

(**A**) NSCs were co-cultured with exosomes for 3 days in differentiation conditions. Representative images of pre-mature neuronal (Tuj1) and glial (GFAP) markers staining were shown. Proportions of cells expressing pre-mature cell-specific immunoreactivities were determined (in the right panel). (**B**) Representative images of matured neuronal (Map2) and glial (Glast) staining were shown. Proportions of cells expressing matured cell-specific immunoreactivities were determined (in the right panel). (**C**) The transcript expression of pre-mature cell markers (*βIII-tubulin* & *GFAP*) and matured cell markers (*Map2* & *GS*) was determined by RT-qPCR analysis. Data were represented as mean ± SE from three independent experiments. * and ** denote p < 0.05 and p < 0.01, respectively. Scale bar 100 μm (**A, B**).


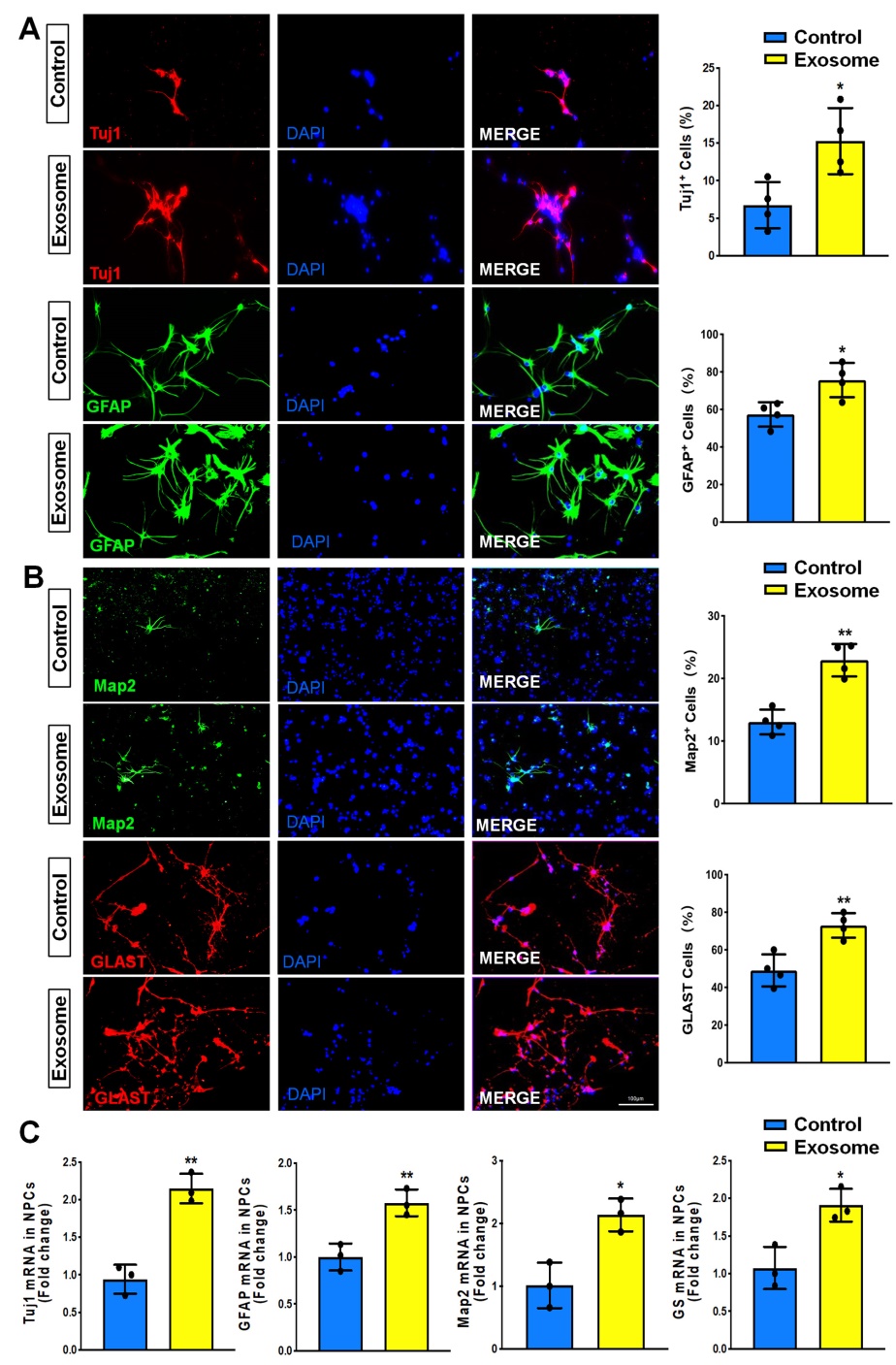


**Supplementary Figure 3. EXOs promote NSC differentiation at culture day 9.**

(**A**) NSCs were co-cultured with exosomes for 9 days in differentiation conditions. Representative images of pre-mature neuronal (Tuj1) and glial (GFAP) markers staining were shown. Proportions of cells expressing pre-mature cell-specific immunoreactivities were determined (in the right panel). (**B**) Representative images of matured neuronal (Map2) and glial (Glast) staining were shown. Proportions of cells expressing matured cell-specific immunoreactivities were determined (in the right panel). (**C**) The transcript expression of pre-mature cell markers (*βIII-tubulin* & *GFAP*) and matured cell markers (*Map2* & *GS*) was determined by RT-qPCR analysis. Data were represented as mean ± SE from three independent experiments. * and ** denote p < 0.05 and p < 0.01, respectively. Scale bar 100 μm (**A, B**).


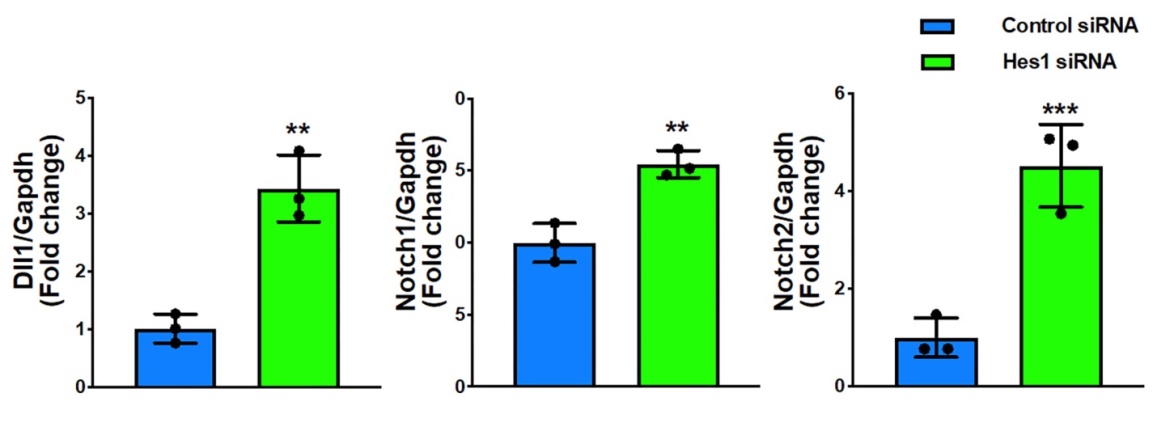


**Supplementary Figure 4. The expression of Notch signaling genes is up-regulated in Hes1 loss-of-function condition.**

The transcript expression of Notch signaling components Dll1, Notch1, and Notch2 in control siRNA- and Hes1 siRNA-transfected NSCs was determined by RT-qPCR analysis. Data were represented as mean ± SE from three independent experiments. ** and *** denote p < 0.01 and p < 0.001, respectively.

| Gene | Sequence | Size(bp) | T^o^ | Accession N. |
| --- | --- | --- | --- | --- |
| *βIII-tubulin*  *(Tuj1)* | 5’-CTTTATCTTCGGTCAGAGTGGTGC-3’  5’-TTCTTTCCGCACGACATCTAGG-3’ | 103 | 57 | NM_023279.2 |
| *Dll1* | 5’-TGCGAAGGAAGCCACTGCAA-3’  5’-GGAAAGGCGTCTCTCGGCTC-3’ | 293 | 57 | NM_007865.3 |
| *Foxg1* | 5’-AGAAGAACGGCAAGTACGAGA-3’  5’-TGTTGAGGGACAGATTGTGGC-3’ | 189 | 58 | NM_005249 |
| *Foxp2* | 5’-TGGCAGCAGGGATGGGAGAT-3’  5’-GGGAGTCATCATGGCCACCG-3’ | 202 | 57 | NM_053242.4 |
| *GAPDH* | 5’-CATGTTCCAGTATGACTCCACTC-3’  5’-GGCCTCACCCCATTTGATGT-3’ | 136 | 60 | NM_001289726.1 |
| *GFAP* | 5’-TTGCTGGAGGGCGAAGAAAA-3’  5’-CATCCCGCATCTCCACAGTC-3’ | 148 | 57 | NM_010277.3 |
| *GS* | 5’-TCACAGGGACAAATGCCGAG-3’  5’-GTTGATGTTGGAGGTTTCGTGG-3’ | 362 | 58 | NM_008131 |
| *Hes1* | 5’- TTTGGCGGCTTCCAAGTGGT-3’  5’- CTCTCAGTTCCGCCACGGTC-3’ | 201 | 57 | NM_008235.2 |
| *Map1b* | 5’-GAGGGAGAGAGGCGGGAGAG-3’  5’-TGCAGACCTGGTTGCTGGTG-3’ | 179 | 57 | NM_008634.2 |
| *Map2* | 5’-AGCCGCAACGCCAATGGATT-3’  5’-TTTGTTCTGAGGCTGGCGAT- 3’ | 313 | 57 | NM_001039934.1 |
| *Msi1* | 5’-CAGGAAGGGCTGCGCGAATA-3’  5’-GGAAAGGCCACCTTGGGGTC-3’ | 194 | 57 | NM_008629.2 |
| *Notch1* | 5’-CTCCACTGATCCTGGCTGCG-3’  5’-TCCCGGTTGGCAAAGTGGTC-3’ | 276 | 57 | NM_145827.4 |
| *Notch2* | 5’-GCCTGTATCAGCAACCCTTG-3’  5’-CCGATCTTATCCAGGCAGGT-3’ | 296 | 57 | NM_010928.2 |
| *Pax6* | 5’-TCCGTTAGAACTGATGGAGT-3’  5’-GTTGGTATCCCGGGACTTC-3’ | 101 | 57 | NM_001310146.1 |
| *REST* | 5’-GTGCGAACTCACACAGGAGA-3’  5’-AAGAGGTTTAGGCCCGTTGT-3’ | 201 | 52 | NM_011263.2 |
| *Tlx* | 5’-TGTGAATCAGCTGCCAGGCTTC-3’  5’-CCAGTAGAGTGTTAGCATCAACCG-3’ | 175 | 57 | NM_152229.3 |
| *Zic5* | 5’-ATGTTCATCTCGGCCAGCGG-3’  5’-CATCTGGCCGTTTAGCGGGT-3’ | 117 | 57 | NM_022987.3 |
| miRNA |  |  |  |  |
| Universal primer | 5’-GAATCGAGCACCAGTTACGC-3’ |  |  |  |
| *U6* | 5’-TGGCCCCTGCGCAAGGATG-3’ |  | 55 |  |
| *let-7f-5p* | 5’-TGAGGTAGTAGATTGTATAGTT-3’ |  | 55 | MIMAT0000525 |
| *let-7g-5p* | 5’-TGAGGTAGTAGTTTGTACAGTT-3’ |  | 55 | MIMAT0000121 |
| *let-7i-5p* | 5’-TGAGGTAGTAGTTTGTGCTGTT-3’ |  | 55 | MIMAT0000415 |
| *miR-129-5p* | 5’-CTTTTTGCGGTCTGGGCTTGC-3’ |  | 55 | MIMAT0000209 |
| *miR-129b-3p* | 5’-CAAGCCCAGACCGCAAAAAGATT-3’ |  | 55 | MIMAT0029863 |
| *miR-21a-5p* | 5’-TAGCTTATCAGACTGATGTTGA-3’ |  | 55 | MIMAT0000530 |
| *miR-30a-5p* | 5’-TGTAAACATCCTCGACTGGAAG-3’ |  | 55 | MIMAT0000128 |
| miR-30e-5p | 5’-TGTAAACATCCTTGACTGGAAG-3’ |  | 55 | MIMAT0000248 |
| *miR-9-5p* | 5’-TCTTTGGTTATCTAGCTGTATGA-3’ |  | 55 | MIMAT0000142 |
| *miR-99a-5p* | 5’-AACCCGTAGATCCGATCTTGTG-3’ |  | 55 | [MIMAT0000131](http://www.mirbase.org/cgi-bin/query.pl?terms=MIMAT0000131) |

**Supplemental Table 1. List of specific primers.**
